# “What does not kill you… mutates and tries again.” A study on personality determinants of post-traumatic growth during the COVID-19 pandemic

**DOI:** 10.1007/s12144-023-04415-5

**Published:** 2023-03-10

**Authors:** Serena Petrocchi, Sara Angela Pellegrino, Greta Manoni, Giada Petrovic, Peter J. Schulz

**Affiliations:** 1 Faculty of Biomedical Sciences, Università della Svizzera italiana, Via Buffi 13, Lugano, 6900 Switzerland; 2 Faculty of Communication, Science, and Society, Università della Svizzera italiana, Via Buffi 13, Lugano, 6900 Switzerland; 3 Department of Communication & Media, Ewha Womans University, Seoul, Republic of Korea

**Keywords:** COVID-19, Post-traumatic growth, Trauma, Sense of control, Well-being, Self-mastery

## Abstract

**Introduction**. The COVID-19 pandemic was recognized as a collective trauma and as a major threat to mental health. Recent literature focused on the stress symptomatology or post-traumatic stress disorder associated to the COVID-19 exposure. The concept that people have a natural inclination toward growth, even under stressful and threatening events, gathered less attention. Previous research has analyzed antecedents of post-traumatic growth (PTG) with non-conclusive results. **Methods**. The present research aimed at including findings on PTG from personality traits, i.e., sense of control and self-mastery, and distal condition of nurturance and support received by others, i.e., cognitive and affective well-being. Analyses were based on 4934 interviews with adults (M_age_ = 57.81 years, 55.5% women) from the Swiss Household Panel study. **Results**. Relationships over time emerged between sense of control and self-mastery on PTG and worries, measured after two years, via the mediation of cognitive and affective well-being. **Conclusion**. Results come from a large study in a design seldom employed in this type of research and can inform both research and interventions.

## Background

“What does not kill you makes you stronger” says a very popular aphorism, which inspired a fruitful line of research within ‘positive psychology’ (Affleck & Tennen, 1996). Then, after 2019, when the COVID-19 pandemic spread all over the world with its variants (at least four by the time this paper was written), perhaps the motto had better be rephrased into something like “what does not kill you… mutates and tries again”. Indeed COVID-19 was identified as a collective trauma (Masiero et al., 2020) and a major threat to mental health (Brooks et al., 2020; Galea, Merchant, & Lurie, 2020; Salari et al., 2020). Due to the pandemic and the uncertainty that came with it (Petrocchi et al., 2021), a large number of people was socially or physically isolated, and as a consequence, there was an outgrowth of loneliness, insecurity, anxiety, depression, substance abuse, and other symptoms related to stress (Benke et al., 2020; Brooks et al., 2020; Galea et al., 2020; Horesh & Brown, 2020; Rodríguez-Rey et al., 2020; Salari et al., 2020). This was a potentially overwhelming and stressful situation for the general population, which promoted stress symptomatology and, in some cases, post-traumatic stress disorder (Dutheil et al., 2021; Karatzias et al., 2020; Koliouli & Canellopoulos, 2021; Shevlin et al., 2020). Traumatic situations are known to have a negative impact on mental health.

In spite of the attention to the negative consequences of traumatic events, there is also research that underlined the possibility of an individual’s positive development even under stressful conditions (Calhoun & Tedeschi, 2006; Tedeschi et al., 2018). The concept that people have resources to stay healthy in the face of difficulties and stressful events (Antonovsky, 1979) presumes that humans have a natural inclination toward growth and development that allows them, under several circumstances, to realize their potentials and seek new challenges. The seminal work by Tedeschi and Calhoun (Calhoun et al., 2010; Calhoun & Tedeschi, 2006; Tedeschi & Calhoun, 2004) emphasized that individuals after trauma and adverse experiences may show gratifying changes that they called post-traumatic growth. Post-traumatic growth induces a qualitative change that leads to positive modifications in the self-perception, interpersonal relationships, and life philosophy (Tedeschi et al., 2018). Post-traumatic growth arises with the efforts to adapt to, and cope with negative events (Tedeschi & Calhoun, 2004) that produce high levels of psychological distress. Making experience of stress, and eventually post-traumatic stress symptomatology, is an important prerequisite for a post-traumatic growth to exist (Celdrán et al., 2021). Among the antecedents of post-traumatic growth, Tedeschi and colleagues (Tedeschi et al., 2018) identified personality traits, ability to manage distressing emotions, proximal and distal conditions of nurturance, and support received by close others.

Research on this field usually focused on trauma survivors, such as cancer survivors (Brivio et al., 2021; Casellas-Grau et al., 2017; Lelorain et al., 2010; Tremolada et al., 2016) and witnesses of traumatic events (First et al., 2017; Fredrickson et al., 2003; Lieberman et al., 2018; Nakagawa et al., 2016; Wen et al., 2020). The COVID-19 pandemic represented a quite unprecedented natural life-threatening event that stimulated a bunch of research on post-traumatic growth in the general population.

Several studies examined the PTG during the COVID-19 pandemic. These studies have suggested that females, younger, more educated, and those living with a partner reported higher rates of post-traumatic growth (Kalaitzaki, 2021; Northfield & Johnston, 2021; Prieto-Ursúa & Jódar, 2020). Although another study (Arnout & Al-Sufyani, 2021) found high level of post-traumatic growth in individuals in the age range of 40–60 and no differences in PTG due to the educational level. Others studies suggested that perceived support from family and friends had a positive effect on post-traumatic growth during the pandemic, directly (Hyun et al., 2021; Liu et al., 2020; Xie & Kim, 2022) or via the moderation effects on the relationship between distress and post-traumatic growth (Northfield & Johnston, 2021). Among the personality factors, some studies (Feng et al., 2021) found that introversive personality was a negative predictor of post-traumatic growth, while the positive effect of extraversion on PTG was observed. Other personality traits positively related to growth are emotional stability, agreeableness, and conscientiousness (Xie & Kim, 2022). Lau and colleagues (2021) discovered that the combination of high levels of post-traumatic stress and sense of coherence determined high levels of post-traumatic growth. Other features, such as beliefs about a good world, openness to the future, and identification with humanity were linked to post traumatic growth during the COVID-19 pandemic (Vazquez et al., 2021). Finally, Matos and colleagues (2021) found that compassion and social safeness predicted higher post-traumatic growth.

With few exceptions (Lau et al., 2021), the research summarized above follows a cross-sectional design, and evidence on the causal links between variables cannot be conclusive. To redress this gap, the present paper aimed to study post-traumatic growth in a sample of people exposed to the COVID-19 pandemic during 2020, considering the effect of sense of control, as a personality trait, measured two years before, and taking into consideration the mediating role of subjective well-being, as a distal condition of nurturance and support received by close others.

## Sense of control and post-traumatic growth

Sense of control refers to the capacity to see themselves as capable to defeat and stand up against adverse events through personal achievement, individual problem solving, and personal capability (Pearlin et al., 1981). In other words, sense of control is one’s perception of being able to achieve a success or to overcome a challenging situation through individual efforts. Meanwhile, a trait-like sense of control can be described as a decisional capacity of the self to adjust adequately and to adapt to the overall context (Tangney et al., 2004). A recent meta-analysis (Ridder et al., 2011) surveying over 100 studies affirmed the advantages of trait-like sense of control in several social settings (i.e., work, school, interpersonal relationships) that generates a better management over one’s urge or behaviors. Moreover, individuals with low self‐control are often involved in a broad range of misbehaviors, including unhealthy eating (Hofmann et al., 2009), lack of exercising, academic failure and underachievement (Duckworth & Seligman, 2016), procrastination, substance abuse, impulse buying (Vohs & Faber, 2007), antisocial behaviors and bullying (Filipponi et al., 2020), and delinquent behavior (Gottfredson, 1990; Moffitt et al., 2011; Patton et al., 1995).

According to Pearlin (2010), a sense of control makes people confident in the ability to manage even negative events that may threaten their life. In other words, the sense of control is an adaptive strategy to lower the impact of stressful situations. Numerous studies have explored how people’s sense of control helps individuals to achieve a resistance against stress (Pearlin et al., 1981; Skinner, 1996). In general, individuals who are endowed with a sense of control are less negatively impacted by stressful life circumstances and therefore they can confront challenges more successfully than those who are doubtful about their capacity to manage their stress successfully (Bandura, 1997; Schwarzer, 1992). It has been proposed that this is, in part, the result of their current efficacy in problem-solving and their objective capabilities to overcome difficulties (Bandura, 1997), and, in part, the outcome of a sustained set of generalized beliefs or perceptions of their own abilities (Bandura, 1997; Carver & Scheier, 1998).

Another way to conceptualize the sense of control is through the concept of self-mastery that is the perception to be competent and worthy, and the confidence to be able to reach desirable outcomes via personal effort (Skinner, 1996; Windsor and Anstey 2009). A high level of perceived control is associated with better cognitive functioning (Agrigoroaei & Lachman, 2011; Infurna & Gerstorf, 2013; Windsor & Anstey, 2009), mental health (Lang & Heckhausen, 2001; Windsor & Anstey, 2009), and physical health (Infurna et al., 2013; Turiano et al., 2014). Thus self-mastery improves a person’s health, helps to avoid harmful behaviors (Manne et al., 2006), and negative thoughts about oneself (Aspinwall & Richter, 1999).

Based on this evidence, we formulated the following hypotheses:

### Hypothesis 1a (HP1a)

sense of control is positively associated with post-traumatic growth and negatively associated with worries about COVID-19.

### Hypothesis 1b (HP1b)

self-mastery is associated with higher post-traumatic growth and lower levels of worries for COVID-19.

## The mediating role of subjective well-being

The sense of control and self-mastery might also be linked to post-traumatic growth indirectly via the mediation of subjective well-being, which is described as the overall positive evaluation of life and emotional experiences (Diener, 1984). Subjective well-being is frequently used to assess the individual’s quality of life (Chang et al., 2019). It comprises the evaluation of one’s life according to a standard individual developed for him/herself. Subjective well-being indicates something like tension between the prospects of one’s life as it is, and as it should be (Chang et al., 2019). Subjective well-being can be split into affective and cognitive well-being. The affective well-being is the type and frequency of positive and negative affect that people experience. Cognitive well-being represents a person’s general evaluation about his/her life.

Research has found that trait sense of control is a key predictor of both cognitive and affective well-being (Briki, 2018; Briki & Majed, 2019; Ridder & Gillebaart, 2016; Hofmann, Luhmann, Fisher, Vohs, & Baumeister, 2014). De Ridder and Gillebaart (2016) hypothesized that people with a high sense of control have no need to restrain their impulses and could engage in more goal-directed activities, which would bring them closer to pursue the objectives they set for themselves, including their emotional balance and life satisfaction. The authors in addition stated that reaching a goal could “constitute an important part of experiencing more well-being since goal achievement has been known to cause positive affect” (Ridder & Gillebaart, 2016, p.93). On the other side, the sense of control is also linked to affective well-being because the principle that a high sense of control makes people to minimize or escape bad feelings and promote good ones or even because high controlled individuals behave appropriately, which thereby reduces stress, guilt, and other bad feelings (Diener et al., 1999).

Affective well-being is also connected to the evaluation of one’s emotions as pleasant or unpleasant, and to experience more positive than negative emotions (Diener et al., 1999; Diener & Ryan, 2009). High affective well-being is characterized by a high level of positive affect, such as happiness, contentment, joy, energy, and relaxation (Diener & Ryan, 2009). According to Fredrickson’s broaden-and-build theory, positive affect is the most important mechanism for individuals to recover from stress (Fredrickson, 2013). In the Hamama & Sharon’s study (Hamama & Ronen-Shenhav, 2012) on Israeli caregivers of hospitalized patients with chronic diseases, caregivers’ positive affect was found to predict their posttraumatic growth. This finding corroborated the earlier report by Linley & Joseph (Linley & Joseph, 2004), which reviewed 39 empirical studies documenting significant relationships between greater positive affection and greater posttraumatic growth. Also, a more recent study indicated that positive affect correlated with high levels of post traumatic growth (Teodorescu et al., 2012). Affective well-being promotes higher self-esteem, perception of hope, and a better sense of meaning, and it decreases personal distress such as anxiety and depression (Gilman & Huebner, 2006; Marques et al., 2011; Nadeau et al., 2015).

These considerations taken together suggest that affective well-being can serve as a mediator in the relationship between sense of control and post-traumatic growth. Therefore, persons who have a sense of control feel affectively well and, in turn, they are likely to succeed in confronting stressful situations such as COVID-19.

According to Calhoun et al. (Calhoun et al., 2010), cognitive well-being would also promote post-traumatic growth. After post-traumatic stress, the way the individual understands the world frequently change. Individuals who reconsider basic assumptions about who they are, what the people around them are like, and what kind of world they live in, make growth possible (Henson et al., 2021). Indeed, post traumatic growth differs from resilience and recovery because it is not merely the restoration of a person’s pre-trauma state of functioning, but is a positive change in previous ways of thinking, indicative of a reorientation of values or priorities (Muldoon et al., 2019). For example, the presence of intentional rumination after the occurrence of a traumatic event is evidence of cognitive wellbeing as well as a process of sense-making and reconstruction of one’s representations of the world (Henson et al., 2021). Other evidence of the relationship between cognitive wellbeing and PTG comes from another research (Triplett et al., 2012) in which authors found that challenging core beliefs correlated with life satisfaction. This evidence suggests that cognitive wellbeing can be another mediator in the relationship between sense of control and posttraumatic growth.

Therefore, based on this evidence, we formulated the following hypothesis:

### Hypothesis 2 (HP2)

affective and cognitive well-being mediate the relationship between sense of control and self-mastery and post-traumatic growth and worries of COVID-19.

**Fig. 1:**
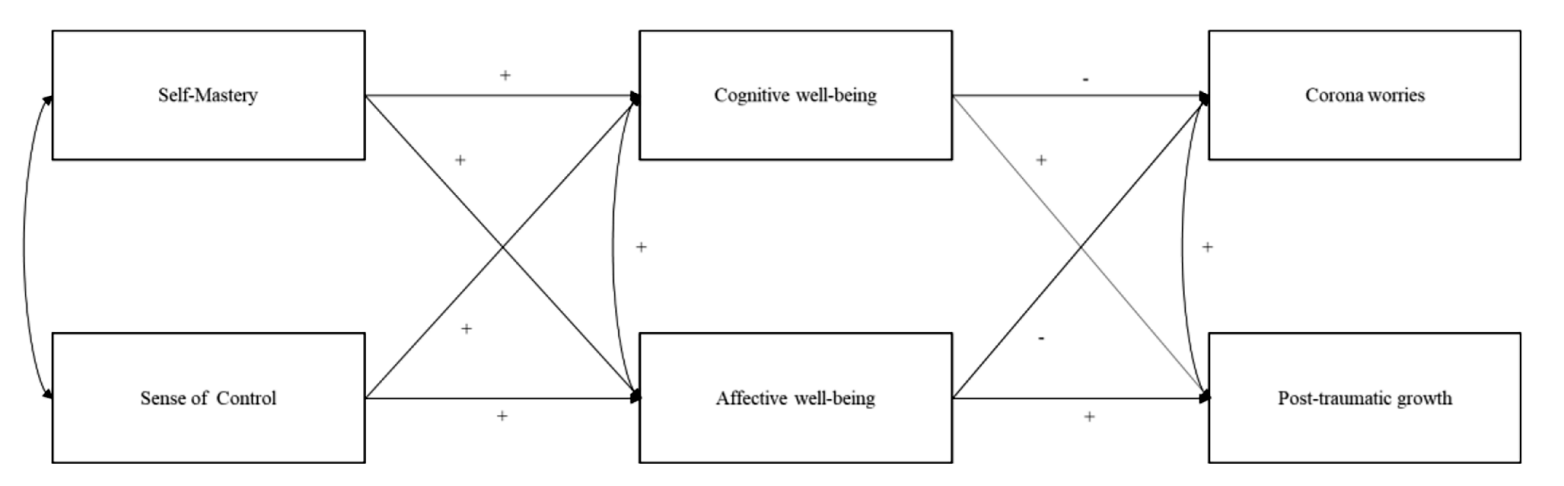
Theoretical model tested

The full theoretical model tested is represented in Fig. 1.

## Methods

### Research design

The present study carried out a secondary data analysis on data sets derived from the Swiss Household Panel (SHP) (Tillmann et al., 2016), a large annual household panel study. The original SHP data collection was based on a stratified random sample conducted in Switzerland since 1999. At present, the SHP (Voorpostel et al., 2017) comprises three cohorts: the SHP_I (5,074 households and 7,799 individuals interviewed from 1999), the SHP_II (2,538 households and 3,654 individuals interviewed from 2004), and the SHP_III (3,989 households and 6,090 individuals interviewed from 2013 to present). In 2020 a specific questionnaire related to COVID-19 was administered in May-June to 5843 Swiss citizens who were partly included in the previous waves. More information about the SHP can be found here Tillmann et al. (2016) and Voorpostel et al. (2017).

The present study took advantage of two different data sets derived from the SHP. The first has been collected in 2018 and includes the so considered independent variables, such as self- control, self-mastery, and wellbeing. This first data set consists of a sample of Swiss participants who mostly replied to a phone interview in 2018. Only a small % (< 10%) of the participants were contacted face-to-face and/or via web link. More information about the 2018 survey can be found here (Voorpostel et al., 2018). The second data set has been collected in May and June 2020 during the mandatory confinement at home as a public health measure against the COVID-19 pandemic. The 15-minute survey was self-administered using web and paper questionnaires. This data set consists of participants who were included in the 2018 data set and participated in the 2020 data collection as well. More information about the 2020 survey can be found here (Refle et al., 2020). The original SHP is based on a stratified random sample of individuals living in Switzerland. However, since the matching of the two data sets has been made considering the data set of the 2020 first, the sample derived for the analysis of the present study cannot be considered at random.

### Procedure

Each household has received information regarding the research project and the procedure before participating in the data collection. Participation is voluntary and participants are informed about the possibility of withdrawing at any moment. Data collection is completely anonymized. To match the subjects’ responses in each wave, participants have been provided with a number code (i.e., ID). The authors of the present study do not have access to the correspondence between the IDs and the participants’ identifying names/surnames. According to the authors of the panel data, the SHP does not fall under the law on research on human beings; therefore, according to the local rules and regulations, it does not require ethical approval. Data collection followed the ethical standards defined by the Declaration of Helsinki. The panel data is freely available. The authors of the present study have the right to use the data since they have signed a contract before downloading it in a completely anonymized form.

### Study participants

In the present study, we included data collected during 2018 (SHP_wave 20) and 2020 (SHP_COVID wave 22) henceforth T1 and T2. The sample in the two time slots were matched starting from the T2 data set. The individuals are the same in the two data sets and can be anonymously identified via a personal ID that remains the same over time. The data of the individuals in the T2 data set were then matched to their data collected at T1, according to their ID. The only exclusion criterion was being over 18 years old. The initial sample in 2020 was composed of 5843 individuals (M = 54.17 years, SD = 18.57 years, range 14–99 years); 293 participants were excluded from the analytical sample because they were under 18 years of age in 2018 (T1), while other 616 participants were excluded because they did not participate in the data collection in T1 (2018). Participants with clinical diagnosis in 2020 (N = 148) have been eliminated from the analytical sample.

The analytical sample was then composed of 4934 individuals (M = 57.81 years; SD = 16.01; range 22–99; 2737 women [55.5%]). Mean of years until the highest educational degree was 14.19 (SD = 3.07; range 8–21), corresponding to the High School Diploma measured at T1 and based on the ISCED-classification scheme (Unesco, 2012). The household size at T2 varied from 1 (n = 1005, 20%), to 2 individuals (n = 2249, 45%), to three or more individuals (n = 1675, 33%). At T2, 55% of the participants were employed, 43% inactive (i.e., in education, retired, stay-at-home parent/partner, unable to work for disability), and 2% unemployed. 55% of the participants were not infected by COVID-19 and did not know anyone infected before May-June 2020; 45% had the infection or knew someone who was infected. 32% of the participants considered themselves to be at risk of complications in case of infection (i.e., because of their age or a pre-existing disease).

## Measures

### Sense of control

was measured at T1 with 6 items: “I can do just about anything I really set my mind to”; “When I really want to do something, I usually find a way to succeed at it”; “Whether or not I am able to get what I want is in my own hands”; “What happens to me in the future mostly depends on me” (Pearlin & Schooler, 1978) and “Other people determine most of what I can and cannot do”; “I sometimes feel I am being pushed around.“ (Lachman & Weaver, 1998). Response options ranged from 0, “I completely disagree”, to 10, “I completely agree”. The scores of the negatively worded items were reverse-coded. A final score was created by calculating the mean of the items, with higher scores indicating higher sense of control (ω = 0.83, α = 0.74, rs > 0.33).

### Self-mastery

was measured at T1 by a 6-item scale as developed by Levy and colleagues (Levy et al., 1997; Strodtbeck, 1958). The scale is composed of three items measuring self-mastery (“I feel like I have little influence on the events of my life”; “I am easily overcome by unexpected problems”; “In general, I have no difficulty choosing between two possibilities”) and two items coming from the self-esteem scale by Rosenberg (Rosenberg et al., 1995) (“Sometimes I feel useless”; “Finally, I am rather pleased with myself”). Range of response options, treatment of negatively worded items, and the computation of a final score were done similar as described for the sense of control measure. Higher scores indicated higher self-mastery (ω = 0.74, α = 0.61, rs > 0.26).

### Subjective well-being

was measured at T1 and was composed of a cognitive and an affective dimension. The cognitive dimension evaluates the general dimension of life satisfaction with 4 items (Diener et al., 1985). Participants were asked to say if agree with the following statements: “In most ways my life is close to my ideal”, “The conditions of my life are excellent”, “So far I have gotten the important things I want in life”, “If I could live my life over, I would change almost nothing”. The affective dimension measures positive and negative affects (Diener, 2000; Diener et al., 1999), such as anxiety, optimism, joy, anger, sadness, and worry. Range and labels for response options, treatment of negatively worded items, and the computation of final scores for cognitive and affective well-being were done similar to the way described for the other measures (cognitive well-being, ω = 0.86, α = 0.83, rs > 0.57; affective well-being, ω = 0.85, α = 0.76, rs > 0.34). Higher scores indicated higher well-being.

### Worries

related to COVID-19 at T2 were measured by a 9-item scale (see Kühne et al. 2020). Participants were asked to say how concerned they are about the following: the economy in general; their own financial situation; their health; the health of their loved ones; whether they will receive the necessary medical treatment if they do contract the coronavirus; social cohesion; their social relationships; their lifestyle; share prices and other forms of investments. Range and labels for response options, treatment of negatively worded items, and the computation of final score were done similar to the way described for the other measures (ω = 0.86, α = 0.80, rs > 0.27).

### Post-traumatic growth

from the Posttraumatic Growth Inventory (Tedeschi & Calhoun, 1996) were applied at T2. The four items asked respondents to indicate the degree of change that had occurred because of the Corona crisis as following: “I established a new path for my life”, “I know that I can handle difficulties”, “I changed my priorities about what is important in life”, and “I have a stronger spirituality/religious faith”. Range and labels for response options, treatment of negatively worded items, and the computation of final scores for cognitive and affective well-being were done similar to the way described for the other measures (ω = 0.77, α = 0.73, rs > 0.42).

### Socio-demographics and other covariates

Information on age, gender, household size, employment status, satisfaction with financial situation during the COVID-19 emergency, and health status at T2 have been collected. Details on the education level based on the ISCED classification (Unesco, 2012), satisfaction with life, and satisfaction with personal relationships have been collected at T1. Then, participants replied to questions on whether they and a relative, friend, or co-worker, have been exposed to the COVID-19 infection (response options: yes/no). They also answered on whether they are included in at-risk group for complications following the COVID-19 infection (response option: yes/no/do not know).

## Data analysis strategy

Preliminary data analyses were conducted in SPSS v.26. First, the data were screened for missing values. Missing data ranged from 0.5 to 1.9%. Next, univariate distributions (i.e., skewness and kurtosis) were examined. All the variables had skewness and kurtosis < ± 0.8, apart from cognitive well-being (Kurtosis = 2.5, Skewness = -1). Bivariate associations between the main variables and the covariates (age, gender, COVID-19 exposure, being part of a risk group for COVID-19, general health, education, household size, and employment status) were calculated with Pearson’s r, Spearman’s rho, or Kendall’s Tau according to the variable. VIF and tolerance have been calculated to detect multicollinearity between variables.

All the subsequent analyses were carried out in R using the Rstudio software v.1.2.5019 and the Lavaan package (Rosseel, 2012). A post-hoc power analysis for the main (second) tested model was calculated applying the semPower package (Jobst et al., 2021) for RStudio. Post-hoc power analysis has been chosen because the present paper is based on a secondary data analysis of the SHP data and the sample size could not be established a-priori. The post-hoc power analysis was therefore calculated with the given sample (N = 4934), and with RMSEA ≥ 0.050, α = 0.05, df = 5, and p = .001.

The partial non-normality of univariate distributions was handled with the Weighted Least Square Mean and Variance method (WLSMV), a robust estimator, which does not assume normally distributed variables. A CFA was used to examine whether the proposed unidimensional factor structure of the scales had good fit to the data. According to Byrne (2010), a CFA model can be accepted when the χ^2^-value is non-significant. However, on large samples (400 cases or more) the χ^2^-value is highly likely to be significant. Therefore, we also considered the following goodness-of-fit indices: CFI > 0.90, RMSEA < 0.08, and SRMR < 0.08, NNFI, NFI, TLI, IFI, and GFI > 0.90 (Hu & Bentler, 1999). Since the measures of self-mastery and sense of control have been derived from a combination of items, the EFA was also applied.

The main expected model was first tested with all the covariates (first model) and then with only significant covariates (second model). Bootstrap resampling distribution based on 1000 resamples was calculated as well as 95% confidence intervals. Because of that, we set the significance level of each statistical test as p < .01 to reduce type II errors. The confidence intervals seemed to be quite tight meaning that the beta values can be trusted. The Δχ^2^, ΔCFI, and ΔRMSEA were calculated to establish what is the best model between the two and R^2^ as well. A non-significant Δ χ^2^ suggests the first model is the best fit of the data, but since the Δχ^2^ depend on sample size, we used also the ΔCFI (i.e. change in CFI) paired with ΔRMSEA (i.e. change in RMSEA) as suggested by several authors (Chen, 2008; Cheung & Rensvold, 2002). A combination between ΔCFI values smaller than or equal to 0.01 and ΔRMSEA smaller than 0.015 suggest the first model is the best fit of the data.

## Results

### Preliminary results

Descriptive statistics for the compound scales are shown in Table 1 together with correlations.

**Table 1:** Correlations between the measures

	M (sd)	1	2	3	4	5	6	7	8	9	10	11	12	13
Age	57.83 (16.02)	− 0.040**	0.612**	− 0.157**	− 0.412**	0.127**	0.559**	− 0.175**	0.027	0.027	0.004	0.020	0.000	0.000
Gender (1)	-		− 0.017	− 0.180**	− 0.068**	0.027	− 0.056**	0.015	0.011	0.018	− 0.017	− 0.011	0.018	0.001
Employment status (2)	-			0.218**	0.329**	− 0.120**	− 0.483**	0.182**	− 0.003	− 0.038*	− 0.013	− 0.030*	0.010	− 0.001
Education (3)	14.19 (3.07)				0.105**	− 0.079**	− 0.129**	0.160**	− 0.001	0.007	0.008	− 0.004	0.017	0.003
Household size (4)	-					− 0.089**	− 0.286**	0.129**	− 0.022	− 0.008	− 0.013	− 0.011	− 0.006	0.004
General health (5)	1.72 (0.68)						0.255**	− 0.040**	− 0.003	0.007	− 0.025	− 0.003	0.005	0.010
Risk group (6)	-							− 0.081**	0.019	0.016	0.004	0.003	− 0.016	0.003
Covid-19 exposure (7)	2.94 (2.23)								− 0.033*	− 0.027	0.007	− 0.003	− 0.018	− 0.014
Self-mastery (8)	6.87 (1.22)									0.488**	0.520**	0.391**	− 0.244**	-067**
Sense of control (9)	7.38 (1.21)										0.442**	0.470**	− 0.252**	− 0.035*
Affective well-being (10)	6.95 (1.27)											0.462**	− 0.343**	− 0.086**
Cognitive well-being (11)	7.67 (1.27)												− 0.304**	− 0.059**
Worries (12)	2.99 (1.48)													0.198**
Post-traumatic growth (13)	3.25 (2.27)													

Note: * p < .05; ** p < .01; *** p < .001

The EFAs on the self-mastery and sense of control gave confirmation of the one-factor structure [self-mastery: 34.29% of the variance explained, factor loadings ranging from 0.49 to 0.68; sense of control: 44.62% of the variance explained, factor loadings ranging from 0.46 to 0.79]. The CFAs confirmed the expected one-factor structure for the scales (see Table 2).

**Table 2:** Exploratory Factor Analysis and Confirmative Factorial Analysis of the measures

	χ^2^	df	CFI	RMSEA [HI-LOW]	SRMR	NNFI	NFI	TLI	IFI	GFI	Range of loadings
Self-mastery	173.118***	7	0.950	0.070 [0.061-0.079]	0.047	0.90	0.95	0.90	0.95	0.99	1.000–4.770
Sense of control	94.724***	8	0.982	0.047 [0.039 − 0.056]	0.042	0.97	0.98	0.97	0.98	0.99	0.572–1.171
Affective well-being	206.603***	8	0.970	0.071 [0.063 − 0.080]	0.056	0.90	0.94	0.90	0.94	0.98	1.000–4.455
Cognitive well-being	10.773**	2	0.997	0.030 [0.014 − 0.049]	0.026	0.99	0.99	0.99	0.99	0.99	1.000–1.126
Worries	428.636***	27	0.970	0.059 [0.055 − 0.064]	0.069	0.96	0.97	0.96	0.97	0.99	0.097–1.000
Post-traumatic growth	35.798***	2	0.992	0.062 [0.045 − 0.081]	0.032	0.97	0.99	0.97	0.99	0.99	0.781–1.725

The output of the power analysis gave a probability of 0.99 to detect an effect quantified by an RSMEA of at least 0.50 based on the 4934 participants of the sample. VIF scores ranged from 1.44 to 1.58 and tolerance ranged from 0.63 to 0.69 meaning there is no multicollinearity problems between the main variables.

## Model estimation

**Fig. 2:**
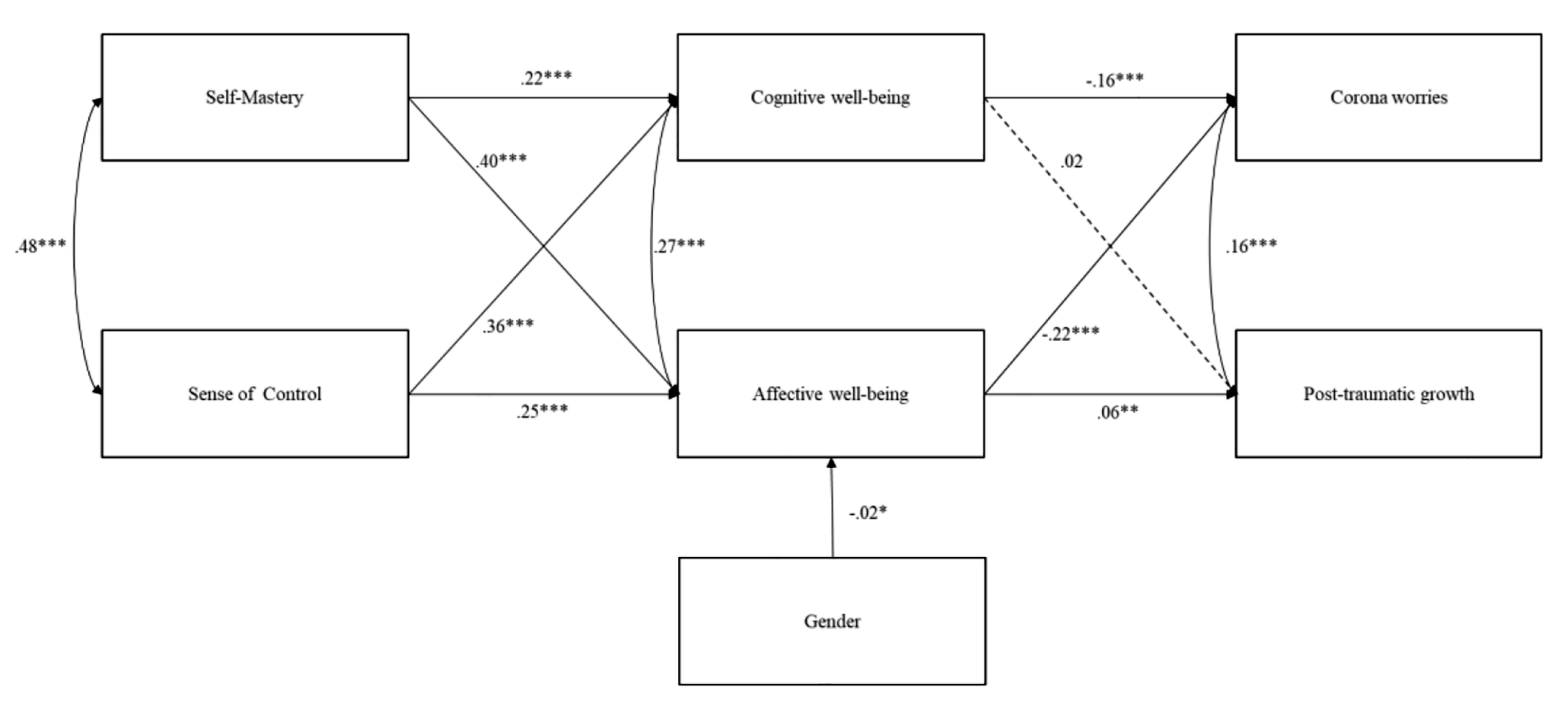
Model tested

Model 1 tested the expected relationships between variables including all the covariates. The model showed nonacceptable fit indices: χ^2^ (27) = 216.49, p < .001, CFI = 0.80, RMSEA = 0.12 (90LO = 0.111, 90HI = 0.14), SRMR = 0.091. The only significant covariates were gender and age; therefore, Model 2 was tested including only those two variables. The model yielded a good fit of the data, χ^2^ (5) = 4.11, p = .53, CFI = 1, RMSEA = 0.00 (90LO = 0.000, 90HI = 0.018, PCLOSE = 1), SRMR = 0.005. Although the change in the chi-square was significant, Δχ^2^ (22) = 212.38, p < .001, the combination of the change in the CFI and RMSEA, ΔCFI = 0.2, ΔRMSEA = 0.12, suggest that the second model is preferable. R-square for post-traumatic growth was 9%, for worries 15%, for cognitive well-being 25%, nd for affective well-being 32%. Figure 2 reports a graphical representation of the paths of the second model.

Paths were significant for self-mastery (*β =* 0.22, p < .001, SE = 0.02, CI95% [0.19 0.26]) and sense of control (*β =* 0.36, p < .001, SE = 0.02, CI95% [0.34 0.41]) considering cognitive well-being. Similarly, the affective well-being increased under the effect of self-mastery (*β =* 0.42, p < .001, SE = 0.02, CI95% [0.38 0.45]) and sense of control (*β =* 0.25, p < .001, SE = 0.02, CI95% [0.22 0.29]). The model also included significant effects of affective well-being (*β =* 0.06, p = .001, SE = 0.03, CI95% [0.05 0.18]) and self-mastery (*β =* 0.04, p = .05, SE = 0.03, CI95% [0.00 0.13]) on post-traumatic growth. Worries at T2 was regressed on cognitive well-being (*β=*-0.16, p < .001, SE = 0.02, CI95% [-0.23 − 0.14]), affective well-being (*β=*-0.22, p < .001, SE = 0.02, CI95% [-0.29 − 0.21]), self-mastery (*β=*-0.04, p = .032, SE = 0.02, CI95% [-0.09 − 0.003]), and sense of control (*β=*-0.07, p < .001, SE = 0.02, CI95% [-0.12 − 0.04]). Gender was associated with affective well-being only (*β=*-0.02, p = .041, SE = 0.03, CI95% [-0.11 − 0.001]). There were significant covariances between worries and post-traumatic growth (*β=*-0.18, p < .001, SE = 0.05, CI95% [-0.64 − 0.44]), affective and cognitive well-being (*β =* 0.27, p < .001, SE = 0.02, CI95% [0.27 0.35]), and self-mastery and sense of control (*β =* 0.48, p < .001, SE = 0.03, CI95% [0.66 0.75]).

The indirect effect of self-mastery on post-traumatic growth through affective well-being was significant (*β =* 0.03, p < .001, SE = 0.02, CI95% [0.02 0.07]), whereas the indirect effect via cognitive well-being was not. The indirect effects of self-mastery on worries through cognitive (*β=*-0.03, p < .001, SE = 0.006, CI95% [-0.05 − 0.031]) and affective well-being (*β=*-0.09, p < .001, SE = 0.01, CI95% [-0.12 − 0.08]) were both significant.

The indirect effects of sense of control on worries through cognitive well-being (*β=*-0.06, p < .001, SE = 0.008, CI95% [-0.09 − 0.05]) and affective well-being (*β=*-0.05, p < .001, SE = 0.007, CI95% [-0.08 − 0.05]) were significant as well as the indirect effect of sense of control on post-traumatic growth through affective well-being (*β=*-0.02, p < .001, SE = 0.009, CI95% [0.01 0.05]). Finally, the indirect effect of sense of control on post-traumatic growth through cognitive well-being was not significant. Total effect for worries was significant (*β=*-0.34, p < .001, SE = 0.02, CI95% [-0.45 − 0.37]) as well as the total effect for post-traumatic growth (*β =* 0.07, p < .001, SE = 0.03, CI95% [0.06 0.20]).

## Discussion

(Petrocchi et al., 2021; Benke et al., 2020; Brooks et al., 2020; Galea et al., 2020; Horesh & Brown, 2020; Rodríguez-Rey et al., 2020; Salari et al., 2020; Dutheil et al., 2021; Karatzias et al., 2020; Koliouli & Canellopoulos, 2021; Shevlin, Hyland, et al., 2020; Shevlin, McBride, et al., 2020)(Calhoun & Tedeschi, 2006; Tedeschi et al., 2018)(Tedeschi et al., 2018). The aim of this paper was to study post-traumatic growth in a sample of people exposed to the COVID-19 pandemic during 2020, considering the effects of sense of control and self-mastery, as personality traits measured two years before, and taking into consideration the mediating role of affective and cognitive well-being. We expected to find that sense of control and self-mastery would be positively associated with post-traumatic growth and negatively with worries for COVID-19. The results are coherent with the hypotheses. The sense of control is the perception of a person’s ability to overcome and resist to stressing events through his efforts (Pearlin et al., 1981; Skinner, 1996). Individuals with a higher sense of control tend to be less negatively touched by stressful life circumstances (Bandura, 1997; Schwarzer, 1992) and overcome challenges more easily (Pearlin et al., 1981), adapting themselves to the overall context (Tangney et al., 2004). Moreover, self-mastery is associated with greater mental and physical health (Infurna et al., 2013; Infurna & Mayer, 2015; Turiano et al., 2014). The sense of control makes people confident in their capability to face negative experiences that could impact their everyday life (Bandura, 1997; Pearlin, 2010; Schwarzer, 1992). For these reasons, individuals with high sense of control and self-mastery may be, during and after difficult moments, more predisposed to grow and less to worries than those with lower levels of sense of control and self-mastery.

We also hypothesized that affective and cognitive well-being would be the mediators in the relationships between sense of control, self-mastery, post-traumatic growth, and worries due to COVID-19. On one hand, results confirmed the hypothesis that self-mastery and sense of control are associated with worries through cognitive and affective well-being. This is in line with previous research focusing on the role of well-being in experiencing more positive than negative emotions (Diener et al., 1999; Oishi & Diener, 2009).

On the other hand, self-mastery and sense of control had indirect relationships with post-traumatic growth via the mediation effect of affective well-being measured through positive and negative affect, such as anxiety, optimism, joy, anger, sadness, and worry as a response of the pandemic. Positive affects generate psychological and interpersonal resources (Fredrickson, 2013), which are known in the literature to facilitate post-traumatic growth. According to Fredrickson’s broaden-and-build theory, positive emotions expand an individual’s instant thought-action repertoire and have three important functions in human life: expanding cognitive perspective, building capacity, and repairing the effects of negative emotions (Altinsoy & Aypay, 2021). Our results demonstrated that emotional stability is one ability to cope with stressful situations (Fteiha & Awwad, 2020) and to provide an adaptive pathway for growth (Park et al., 2008). Our findings are also in line with the results of another recent study (Altınsoy & Aypay, 2021), which found that happiness-increasing strategies (i.e., purposeful activities that an individual uses to maintain and increase happiness) predict post traumatic growth.

Positive and negative emotions influence also cognitive modes of thinking. It is well known that negative emotions influence autonomic nervous systems (Fredrickson et al., 2000; Gross, Fredrickson, and Levenson 1994; Levenson, Ekman, and Friesen 1990). Similarly, positive emotions can undo the persistence of the activations due to negative emotional arousal (Fredrickson et al., 2000; Fredrickson & Levenson, 1998). Therefore, high levels of affective well-being, that is the combination of low levels of negative emotions and high levels of positive emotions, are linked to a well-balanced physiological functioning. Negative emotions narrow individuals’ attention to support attack-or-escape strategies, whereas positive emotions broaden attention, thinking, and behavioral choices (Fredrickson et al., 2000; Fredrickson & Levenson, 1998). Therefore, affective well-being increases flexible, creative, and efficient patterns of thought (Isen et al., 1987; Isen & Means, 1983; Isen, Rosenzweig, & Young, 1991). In this line, under more positive emotional and less negative emotional states, individuals are more likely to broaden their attention and cognitive competence, which in turn facilitates PTG (Fredrickson, 2004; Fredrickson et al., 2003).

One may wonder why cognitive well-being was not found to be related with post-traumatic growth. It should be noticed that cognitive well-being has been measured as a life satisfaction in the present research. The relationship between life satisfaction and post traumatic growth measured during the COVID-19 pandemic was found to be moderated by the severity of the traumatic symptoms (Tomaszek & Muchacka-Cymerman, 2020). These authors found that if the symptoms do not reach high levels of severity, the link between post traumatic growth and life satisfaction is not significant. We might then suggest that the stress in our sample was not high enough to make the relationship between cognitive well-being and growth significant. One motive is that the sampling frame was the general population rather than individuals who were especially affected by the pandemic (e.g., health workers).

In addition, Calhoun and Tedeschi (2014) insisted on the fact that, in order for growth to be possible, traumatic events must have a big enough impact to “force” individuals to reassess their representation of themselves and others, the world they live in, and what the future may bring. It had been empirically demonstrated that core beliefs must be challenged in order for growth to occur (Ramos & Leal, 2013). Our results showed a weak correlation between cognitive well-being and post-traumatic growth, partly because stress levels may be too low in our sample to elicit these processes.

The findings of the present study must be interpreted considering some limitations as well. First, the Swiss Household Panel provided self-report measures only, whereas measures with direct observations have not been included due to the large panel study and the COVID-19 pandemic. Second, the post traumatic growth measurement was derived by the questionnaire developed by Tedeschi and Calhoun (1996). Since not all the original items were used in the Swiss Household Panel data collection in 2020, this measure may not be sufficiently accurate to be considered a complete way to assess PTG. However, as a demonstration of concurrent validity of the PTG measure applied in the present study, there is the positive correlation with worries for COVID-19. As Tedeschi and Calhoun suggested (Tedeschi & Calhoun, 2004), the post-traumatic growth arises together with the distress due to negative events. Moreover, some of the reliability values can be considered as modest. This is a limitation for the reliability of the scales that should be taken into account. Despite the large sample size, the present study presents a limitation in its generalizability to other populations. Finally, in order to make a stronger argument about causal relationships between variables, we should have evaluated the same variables both in T1 and T2. For example, the most suitable research design for this kind of considerations is the RI-CLPM (the random intercept cross lagged panel model). Unfortunately, we could not apply this kind of design in the present study because the SHP does not include in the 2020 the measures collected in 2018.

## Conclusion

The present research demonstrated that personality features, such as sense of control and self-mastery, may be considered as antecedents of post-traumatic growth, as hypothesized by Tedeschi and colleagues (Tedeschi et al., 2018). We also demonstrated that well-being, especially the one linked to balanced emotions, as proximal and distal conditions of nurturance, is mediator in the relationships between personality traits and post-traumatic growth. Our results can inform both research and interventions.

Future research may want to test whether the post-traumatic growth is a long-lasting competence or not. Since sense of control directly, and indirectly, is associated with post-traumatic growth, and as a personality trait it is stable characteristics of individuals, one might ask: how long does the positive influence of personality on growth last? And then how long post-traumatic growth last? Is it a time limited human experience or is it an achievement that can induce other benefits through a virtuous circle? Or else, as someone hypothesized (Maercker & Zoellner, 2004), the post-traumatic growth is a sort of illusion to go through a difficult time? Future research should demonstrate whether the effect of personality traits on growth is stable over a wider timespan and whether the growth is able to reinforce positive outcomes. Therefore, whether the growth is a constructive or illusory process.

Our results may inform interventions as well. According to the model of Baumeister et al. (Baumeister et al., 1994), self-regulation can be exerted like a muscle. It can be reduced after exertion and strengthened through practice. The question is whether sense of control, which is associated with growth, is limited because the amount of resource is finite, or it can be exercised and strengthened as a protective factor to prevent from negative outcomes after a stressful situation. Another important point is than whether growth can be exerted and developed in advanced over a stressful situation, which is not always predictable, as a form of self-help.

## Data Availability

The study has been realized using the data collected by the Swiss Centre of Expertise in the Social Sciences FORS for the Swiss Household Panel Project (SHP). All files are freely available from the ForsBase web page (see https://forscenter.ch/projects/swiss-household-panel/data/). The present paper worked on the data collected in 2018 (the so called: SHP_wave 20) and 2020 (SHP_COVID wave 22).
